# Social Determinants of Health and Access to Orthopedic Care Among Seniors in Little Havana

**DOI:** 10.7759/cureus.104859

**Published:** 2026-03-08

**Authors:** Klaudia Greer, Devon Foster, Jonathan Brutti, Zachary Grand, Blake Padgett, Onelia Lage

**Affiliations:** 1 Department of Medicine, Florida International University, Herbert Wertheim College of Medicine, Miami, USA; 2 Department of Medical Education, Florida International University, Herbert Wertheim College of Medicine, Miami, USA

**Keywords:** access to care, hispanic population, orthopedics, seniors, social determinants of health

## Abstract

Introduction: Little Havana, Miami, Florida, is a predominantly Hispanic neighborhood with many adults and seniors aged 65 and older at risk for osteoarthritis, osteoporosis, and associated fractures. Despite this need, access to orthopedic care is limited by socioeconomic, linguistic, and transportation barriers. This study aims to descriptively characterize community-specific social determinants of health, hip fracture hospitalization rates, and local orthopedic service availability among seniors in Little Havana to identify potential gaps in service availability and inform future research and quality-improvement efforts.

Methods: A descriptive, cross-sectional analysis was conducted using publicly available, aggregate-level data from Statistical Atlas and Miami-Dade Matters. The study population comprised adults and seniors aged ≥65 years residing in six Little Havana zip codes (33125, 33128, 33129, 33130, 33135, and 33136). Variables included insurance coverage, poverty rates, English proficiency, transportation access, hip fracture hospitalization rates, and orthopedic clinic distribution. Data were summarized across zip codes using means and standard deviations and descriptively compared with Miami-Dade County averages. No inferential statistical testing was performed.

Results: Public health data showed that seniors in Little Havana had higher rates of foreign-born status (62.6% ± 10.7% vs. 54.1% countywide) and limited English proficiency (66.5% ± 22.4% vs. 54.1% countywide). The adult uninsured rate was 31.1% ± 9.2%, compared with 21.8% countywide, and 39.8% ± 13.7% of seniors lived in poverty, compared with 21.2% countywide. Transportation barriers were notable, with 23.3% ± 10.1% of households lacking a vehicle. Hip fracture hospitalization rates were substantially higher than county levels (female residents: 462.4 vs. 254.2; male residents: 350.8 vs. 122.5 per 100,000). Only seven orthopedic centers serve the neighborhood, with none in the highest need zip code.

Conclusion: Adults and seniors in Little Havana are disproportionately affected by social determinants of health that may limit access to orthopedic care, including inadequate insurance coverage, lack of transportation, language barriers, and gaps in local service availability. These findings identify key areas where targeted interventions may help reduce disparities and improve orthopedic outcomes in this population, such as expanding insurance coverage, strengthening translation services, improving transportation support, and increasing the availability of local orthopedic care.

## Introduction

Little Havana is a vibrant neighborhood in Miami, Florida, known for its strong Cuban heritage and predominantly Hispanic population. The area encompasses six zip codes (33125, 33128, 33129, 33130, 33135, and 33136) [1]. It is home to about 28,628 seniors aged 65 and older [2]. This demographic is particularly vulnerable to age-related musculoskeletal conditions such as osteoarthritis and osteoporosis, which are associated with impaired mobility, increased fall risk, and reduced bone strength, thereby substantially increasing the risk of hip fractures among older adults [3,4]. Management of hip fractures depends on timely access to coordinated orthopedic care, including surgical intervention and postacute rehabilitation.

However, residents of Little Havana may be disproportionately affected by social determinants of health that influence access to orthopedic care. Existing research has demonstrated that disparities in musculoskeletal care are widespread in the United States and affect multiple dimensions of orthopedic care, including access to outpatient services, utilization of specialty clinics, and follow-up care [5]. These disparities are associated with factors such as insurance status, race, and socioeconomic disadvantage [5]. For example, in the outpatient setting, insurance status in particular has been shown to significantly affect access to musculoskeletal care, travel burden, and utilization of services, with patients who are uninsured or publicly insured experiencing greater barriers to specialty orthopedic care than privately insured patients [5]. Although these disparities are well-documented, existing literature has largely focused on broad populations, with limited attention to how social determinants of health, orthopedic outcomes, and local orthopedic care availability intersect at the neighborhood level in underserved and elderly Hispanic communities. This study aims to characterize neighborhood-level social determinants of health, the orthopedic injury burden, as reflected by hip fracture hospitalization rates, and the geographic availability of orthopedic care services to identify potential access gaps affecting seniors in Little Havana.

## Materials and methods

Study design

The study was conducted as a descriptive, cross-sectional analysis of aggregate, publicly available data. It focused on the six zip codes that comprise Little Havana, Miami, Florida, as defined by the Statistical Atlas neighborhood overview [1]. This included zip codes 33125, 33128, 33129, 33130, 33135, and 33136. All available population-level data corresponding to residents within these zip codes were included, and no sampling or subsampling was performed.

A cross-sectional analysis of public health data from Miami-Dade Matters, a publicly accessible county-level health data repository maintained by the Health Council of South Florida, was conducted on November 3, 2024, to assess factors related to social determinants of health and orthopedic outcomes in Little Havana [2]. The data reflect the most recent multiyear estimates available in the database at the time of extraction, primarily derived from the American Community Survey five-year estimates and Florida Agency for Health Care Administration hospitalization datasets (2018-2022).

Variables collected included the percentage of the population that was foreign-born, the percentage of seniors (defined as over the age of 65) who had difficulty speaking English, the percentage of adults without health insurance, the percentage of seniors in poverty, the percentage of households without a vehicle, and the hip fracture hospitalization rate. Additionally, a large language model (ChatGPT, OpenAI) was used to identify orthopedic practices within the target zip codes. The model was prompted to generate a list of orthopedic surgery clinics located in the specified geographic region. All suggested clinics were independently verified using practice websites, health system directories, and map-based search tools to confirm the availability of orthopedic services and correct geographic location. For this study, an "orthopedic care center" was defined as a facility primarily offering orthopedic surgery and/or nonoperative orthopedic specialty care (e.g., sports medicine/orthopedics clinics). Physical therapy-only clinics, chiropractic offices, and primary care clinics without orthopedic specialty services were excluded.

Statistical analysis

Variables from the Miami-Dade Matters database were extracted separately for each of the six Little Havana zip codes and entered into Microsoft Excel (Microsoft Corporation, Redmond, WA). For each variable, the six zip code values were averaged to produce a single summary estimate representing Little Havana as a whole, and the standard deviation was calculated to describe variation across zip codes. These Little Havana averages were then descriptively compared with Miami-Dade County averages reported by the same source database.

Because this study was a descriptive, cross-sectional analysis using publicly available aggregate-level data, individual-level data and distributional information were unavailable, precluding formal inferential statistical testing (e.g., t-tests, chi-square tests, regression analyses) and calculation of test statistics or p values for county-level comparisons. The purpose of the analysis was to characterize patterns and disparities in social determinants of health and orthopedic outcomes rather than to test hypotheses or infer statistical significance.

Ethical considerations

This study used publicly available, deidentified data and did not involve human subjects as defined by federal regulations. Therefore, institutional review board approval and informed consent were not required.

## Results

Demographic analysis of the six zip codes comprising Little Havana revealed a senior population of about 28,628 individuals. Public health data showed that 62.6% ± 10.7% of seniors were foreign-born, which is notably higher than the Miami-Dade County average of 54.1% (Table 1). Additionally, 66.5% ± 22.4% of this elderly population reported difficulty speaking English, again exceeding the county average of 54.1%.

**Table 1:** Social determinants of health indicators in Little Havana vs. Miami-Dade county The data collected from Miami-Dade matters [2] are represented as mean percentage (mean %) ± SD when available. For each variable, the six zip code values were averaged (unweighted) to create a neighborhood-level summary estimate. Because zip code population sizes differ, this approach may overrepresent smaller zip codes; future studies should use population-weighted estimates. Outliers were not excluded because all zip codes were prespecified as part of Little Havana; however, variability is reflected in the SDs SD: standard deviation

Little Havana zip code	33125	33128	33129	33130	33135	33136	Little Havana (mean % ± SD)	County (mean %)
Foreign-born	68.5	70.6	57.6	59.4	74.3	45.1	62.6 ± 10.7	54.1
Seniors with difficulty speaking English	83.1	81.2	35.9	80.8	78.6	39.4	66.5 ± 22.4	54.1
Uninsured adults	38.1	40.0	16.6	25.2	37.5	29.1	31.1 ± 9.2	21.8
Seniors in poverty	38.2	57.4	15.4	41.8	41.6	44.6	39.8 ± 13.7	21.2
Households without a vehicle	21.0	35.9	5.6	24.2	24.3	28.8	23.3 ± 10.1	9.7

Healthcare access disparities were also prominent. In Little Havana, 31.1% ± 9.2% of adults lacked health insurance, compared to 21.8% in Miami-Dade County. It was also revealed that poverty disproportionately affected seniors in Little Havana, with 39.8% ± 13.7% of seniors living below the poverty line, compared with just 21.2% across the county. Transportation limitations were evident as well. An average of 23.3% ± 10.1% of households in Little Havana lacked access to a personal vehicle, more than twice the Miami-Dade average of 9.7%.

In terms of orthopedic health outcomes, hospitalization rates for hip fractures among Little Havana seniors were substantially higher than the county average (Table 2). Female residents aged 65 and older experienced 462.4 ± 44.6 hospitalizations per 100,000, while male residents had a rate of 350.8 ± 107.8. These figures far exceed the corresponding county rates of 254.2 and 122.5, respectively.

**Table 2:** Hospitalization rates due to hip fractures among seniors in Little Havana vs. Miami-Dade County The data extracted from Miami-Dade matters [2] are represented as mean ± SD when available data allow ^*^HF hospitalizations per 100,000 female (F) or male (M) residents over the age of 65 SD: standard deviation; HF: hip fracture

Little Havana zip code	33125	33128	33129	33130	33135	33136	Little Havana (mean ± SD)	County (mean)
Senior HF (F)^*^	451.5	461.9	413.3	418.1	512.6	516.7	462.4 ± 44.6	254.2
Senior HF (M)^*^	319.9	485.2	308.4	308.1	208.6	474.3	350.8 ± 107.8	122.5

## Discussion

Access to orthopedic care in underserved communities like Little Havana is shaped by intersecting socioeconomic, linguistic, and cultural barriers. This is supported by the disparities that exist in insurance coverage, interpreter availability, and provider representation across multiple studies [6-9]. Our findings contextualize these well-described barriers at the neighborhood level by demonstrating that Little Havana exhibits a higher burden of adverse social determinants of health than Miami-Dade County averages, with variability across individual zip codes.

Economic barriers and insurance coverage

Orthopedic care access in Little Havana is constrained by significant socioeconomic challenges. Low income and lack of health insurance coverage are well-documented barriers that delay care and are associated with worse access to orthopedic services [5,6]. Consistent with these patterns, our analysis showed that 31.1% ± 9.2% of adults in Little Havana were uninsured, exceeding the Miami-Dade County average of 21.8%, while 39.8% ± 13.7% of seniors lived below the poverty line compared with 21.2% countywide.

Individuals without insurance or with Medicaid often postpone treatment due to cost, contributing to advanced disease and higher complication rates; uninsured or lower income adults with hip osteoarthritis have been shown to have significantly higher odds of being unable to afford or utilize needed healthcare [6]. Access barriers are further compounded by limited acceptance of Medicaid by orthopedic clinics, which may redirect patients to safety-net hospitals or emergency departments for follow-up care [7]. For example, a simulated patient audit in Texas found that only 14% of Medicaid callers could schedule an orthopedic follow-up for a fracture, compared to over 80% of privately insured callers [7].

Within Little Havana, economic disadvantage was not uniformly distributed. Zip code 33128 demonstrated the highest proportion of uninsured adults (40%) and seniors living in poverty (57.4%), highlighting intraneighborhood heterogeneity that may influence access to specialty orthopedic services. These financial barriers are exacerbated by the scarcity of local orthopedic facilities in Little Havana’s highest need areas (e.g., no clinic in zip code 33128), potentially forcing patients to travel outside their community for care. Such travel might increase cost and logistical burden, impeding routine and postoperative follow-up. 

Language and communication barriers

Linguistic isolation represents another major barrier in Little Havana’s predominantly Spanish-speaking community. Limited English proficiency (LEP) interferes with effective communication between patients and providers and is associated with miscommunication and lower quality care. In our study population, 66.5% ± 22.4% of seniors reported difficulty speaking English, notably higher than the Miami-Dade County average of 54.1%, indicating a substantial burden of language-related barriers at the community level.

Many orthopedic practices lack professional medical interpreters or bilingual staff, instead relying on ad hoc interpretation by family members [8], a practice that increases the risk of errors. Studies of orthopedic clinics in South Florida demonstrate limited availability of qualified interpreters, with only 7.7% of clinics reporting access to professional interpreter services [8].

Inadequate interpretation is associated with misunderstandings, diagnostic errors, and poor adherence to treatment plans [10]. Older adults with LEP may avoid or delay care due to fear or frustration related to communication barriers, and Latinx patients with language discordance report lower satisfaction and greater mistrust in healthcare settings [10]. Language discordance has also been linked to worse clinical outcomes following orthopedic injury; older adults with hip fractures who prefer a non-English language have demonstrated higher postoperative complication rates, including delirium and longer hospital stays, despite similar surgical timelines [11]. These findings highlight that language access is a critical component of patient safety and care quality, and that use of professional interpreters and bilingual providers can improve communication, trust, and adherence in orthopedic care settings [8,10].

Cultural barriers and trust in providers

Cultural factors further influence health-seeking behavior and trust in orthopedic care. Patients are often more comfortable with providers who share their language and cultural background, yet Hispanic representation among orthopedic professionals remains low nationwide, with only 2%-3% of practicing orthopedic surgeons identifying as Hispanic or Latino [12,13]. This underrepresentation contributes to limited cultural concordance between providers and patients in communities like Little Havana, which is predominantly Hispanic and has a high proportion of foreign-born residents (62.6% ± 10.7% vs. 54.1% countywide).

Hispanic patients report lower levels of trust and perceived respect from physicians, particularly when prior experiences included discrimination or language barriers [10]. Cultural misalignment may lead to reduced engagement with preventive services, delayed care-seeking, and reliance on alternative health practices if medical advice is not culturally acknowledged. Improving provider diversity and cultural competency has been associated with increased patient trust and satisfaction [14]. Efforts to expand bilingual staffing, cultural sensitivity training, and partnerships with community health workers may help bridge these gaps and encourage earlier engagement with orthopedic services.

Transportation limitations

Transportation poses a significant practical barrier to orthopedic care access in Little Havana, particularly for older adults. Approximately 23% of households lack access to a personal vehicle, more than double the countywide average of about 10%. Limited mobility options make it difficult for seniors with osteoarthritis or healing fractures to attend distant specialty appointments, increasing the likelihood of missed visits and delayed care. Lack of reliable transportation has been associated with poorer chronic disease management and worse health outcomes [15].

These transportation barriers may be particularly consequential in Little Havana, where orthopedic care facilities are unevenly distributed across zip codes, requiring some residents to travel outside their immediate neighborhood for specialty care. Interventions such as nonemergency medical transportation, transportation vouchers, and mobile health clinics have been shown to improve healthcare continuity and service utilization in underserved populations [15,16]. Addressing transportation barriers is, therefore, essential to improving orthopedic care access in Little Havana.

Implications of findings and potential intervention

Given the high hip fracture hospitalization rates observed in Little Havana relative to county averages (Table 2) and the measured barriers (insurance, language, and transportation), interventions that reduce fall risk and improve access to specialty evaluation may be most relevant to this community. Community-based exercise programs focused on balance, strength, and home safety have consistently demonstrated reductions in fall risk of approximately 20%-30% among older adults [17,18].

Nutrition and bone health interventions, including culturally tailored education on calcium and vitamin D intake, have been associated with improved bone health knowledge and modest increases in dietary intake in Hispanic communities [14]. Adequate nutrition combined with weight-bearing exercise can slow bone loss and reduce fracture risk [19]. Public health initiatives such as home safety modifications, community exercise programs, and osteoporosis risk screening may help address upstream contributors to orthopedic injury.

Integrating solutions to reduce disparities

Reducing orthopedic care disparities in Little Havana requires a coordinated, multifaceted approach addressing economic, linguistic, cultural, and transportation barriers. Our findings highlight that these barriers coexist and vary across zip codes within a single neighborhood, underscoring the importance of targeted, community-specific interventions rather than uniform solutions. Expanding insurance coverage, improving local availability of orthopedic services, incorporating interpreter services, and strengthening community partnerships are all essential components [5,8,10,14]. Transportation support and mobile clinic models may further reduce access barriers [15,16]. Evidence from other underserved settings suggests that integrated interventions combining insurance navigation, service availability, and patient education can improve utilization and outcomes [5,16]. Continued community-engaged research is needed to refine these approaches and promote equitable musculoskeletal health.

Limitations of the study

This study has several limitations that should be considered when interpreting the findings. First, we relied on publicly available, aggregate-level data to conduct a cross-sectional analysis, which limits the ability to establish causal relationships between social determinants of health and orthopedic outcomes in Little Havana. Future studies should collect and analyze individual-level clinical data, including comorbidities, fracture mechanisms, and postoperative outcomes, which were not available to us. Furthermore, public data measures such as language proficiency and transportation access are subject to reporting bias. Second, it is possible that hip fractures that were managed nonoperatively, went untreated, or were treated outside the county were not included in the hospitalization rates. Third, the identification of orthopedic care centers using ChatGPT and Google Maps may have misclassified or omitted certain practices, particularly smaller clinics or facilities with limited online presence. Finally, this study focused on a single neighborhood, which may limit generalizability to other Hispanic or underserved communities. Despite these limitations, the findings shed light on barriers to orthopedic care and emphasize areas for targeted intervention and future research.

## Conclusions

Residents of Little Havana experience a high burden of social determinants of health, including elevated poverty rates, language isolation, lack of insurance, limited transportation, and sparse availability of local orthopedic services. In this descriptive, neighborhood-level analysis, these factors co-occurred with higher hip fracture hospitalization rates than Miami-Dade County averages. Although this study does not establish causal relationships, the findings highlight important geographic and sociodemographic patterns that may help contextualize variability in orthopedic care access within underserved Hispanic communities. These results underscore the need for further analytic and longitudinal research to better evaluate relationships between social determinants, orthopedic care access, and musculoskeletal health outcomes. Targeted, community-informed strategies, such as expanding insurance coverage, strengthening local orthopedic service availability, enhancing culturally competent care, and supporting transportation and preventive health initiatives, represent important areas for future intervention and policy consideration to promote equity in orthopedic care access within disadvantaged communities such as Little Havana.
